# Cancer Vaccine Immunotherapy with RNA-Loaded Liposomes

**DOI:** 10.3390/ijms19102890

**Published:** 2018-09-23

**Authors:** Elias J. Sayour, Hector R. Mendez-Gomez, Duane A. Mitchell

**Affiliations:** Preston A. Wells, Jr. Center for Brain Tumor Therapy, University of Florida Brain Tumor Immunotherapy Program, Lilian S. Wells Department of Neurosurgery, 1149 South Newell Drive, McKnight Brain Institute, University of Florida, Gainesville, FL 32611, USA; Hector.MendezGomez@neurosurgery.ufl.edu (H.R.M.-G.); Duane.Mitchell@neurosurgery.ufl.edu (D.A.M.)

**Keywords:** immunotherapy, liposomes, RNA, cancer vaccines

## Abstract

Cancer vaccines may be harnessed to incite immunity against poorly immunogenic tumors, however they have failed in therapeutic settings. Poor antigenicity coupled with systemic and intratumoral immune suppression have been significant drawbacks. RNA encoding for tumor associated or specific epitopes can serve as a more immunogenic and expeditious trigger of anti-tumor immunity. RNA stimulates innate immunity through toll like receptor stimulation producing type I interferon, and it mediates potent adaptive responses. Since RNA is inherently unstable, delivery systems have been developed to protect and deliver it to intended targets in vivo. In this review, we discuss liposomes as RNA delivery vehicles and their role as cancer vaccines.

## 1. Introduction

Cancer immunotherapy is a burgeoning field with evidence for anti-tumor activity in several randomized phase III trials [1,2,3,4]. Much of the excitement surrounding this technology is centered on the concept of immune checkpoint blockade where T cell co-inhibitory signals are antagonized with monoclonal antibodies (mAbs) [5,6,7]. However, immune checkpoint inhibition appears to work predominantly in cancers with high mutational burdens [8,9,10]. Evidence suggests that a preexisting T cell response is required for a response to immune checkpoint blockade, which could be enriched in mutation rich tumors [10,11]. If this is true, the utility of checkpoint inhibitors could be tempered for many cancers with low mutational burdens or immunologically poor tumor microenvironments. To broaden the response to immunotherapy, cancer vaccines may be harnessed to incite response against poorly immunogenic tumors; however they have failed in therapeutic settings [12]. Poor antigenicity coupled with systemic and intratumoral immune suppression have been significant drawbacks [12]. RNA encoding for tumor associated or tumor specific epitopes can serve as a more immunogenic and expeditious trigger of anti-tumor immunity. Since RNA is inherently unstable, delivery systems have been developed to protect and deliver it to intended targets in vivo. In this review, we discuss liposomes as RNA delivery vehicles and their role as cancer vaccines.

## 2. Cancer Vaccines

Cancer vaccines can be utilized to induce de novo responses against tumor specific antigens. Prototypical vaccines are typically composed of antigen coupled with an adjuvant before local administration [13,14,15]. Local administration releases damage associated molecular patterns (DAMPs), leading to a cascade of innate inflammatory mediators and release of a chemokine gradient for chemotaxis of antigen presenting cells (APCs) [16,17,18,19]. APC chemotaxis toward the site of local inflammation allows these cells to pick up antigen before migrating to draining lymph nodes where they present to and prime an activated T cell response against tumor specific antigens [19]. In the setting of prophylactic infectious disease vaccines, this process needs to be continually boosted over months to years to maximize response and immunologic memory therein [20]. In the naïve state, the immune system can be reprogrammed slowly over time with vaccines. In the setting of active malignancy, however, the immune system is edited from an immune activated state to a regulatory milieu that is overcome with profound immunosuppression [21]. Moreover, in the malignant state, cancers are actively evolving as dynamic entities that may not respond to a static vaccine. Even with an appropriate response, in the absence of a minimal residual disease state, aggressive malignancies often spread so rapidly that patients may not have the appropriate amount of time to develop a fully-fledged anti-tumor immune response that is robust enough to overcome gross disease burden [20,21]. Unlike prophylactic infectious disease vaccines which require serial injections over months to years in immune-replete children, anti-cancer vaccines must elicit immunologic activity more rapidly in immunocompromised patients often after or concomitant with cytotoxic chemotherapy [22,23,24]. This is a significant challenge that is highlighted by the HPV (human papillomavirus) vaccine’s failure (although successful at preventing cervical cancer in the prophylactic setting) to induce anti-tumor efficacy in the therapeutic setting [25]. The immunostimulatory capacity of these vaccines may be insufficient in therapeutic contexts, as they require multiple boosts over many months to years to confer appropriate protection [20]. In addition, a fleeting immune response elicited by cancer vaccines might be quickly overwhelmed by tumor induced immunosuppression, both locally and systemically [26,27,28,29,30,31,32,33]. For cancer vaccines to have a niche, new technologies need to be developed to harness the immune system in a personalized and directed manner against tumor specific antigens.

Most prototypical vaccines involve peptides and tend to have poor immunogenicity, however they can be bound to adjuvants (i.e., granulocyte-macrophage colony-stimulating factor, keyhole limpet hemocyanin, aluminum) to confer an immunologic response [34,35,36]. Unlike peptides, nucleic acids are immunogenic without the need for adjuvant; however, DNA vaccines have suffered from poor immunogenicity and require traversion across both cell and nuclear membranes [37,38]. To develop a more robust and facile vaccine, we and others have prioritized mRNA as a more optimal source of tumor specific antigens [38,39,40]. Cells have evolved with rigorous methods (pathogen recognition receptors such as toll-like receptors (TLRs) and intracellular sensors such as retinoic acid-inducible gene-I and melanoma differentiation antigen 5) to recognize RNA as foreign [41,42,43,44,45]. While mRNA is routinely generated by cells for translation, its processing is tightly regulated [46,47]. Yet, when RNA is introduced into a cell externally, as may occur during a viral infection, this stimulates an innate cellular immune response [48,49]. Single stranded RNA sensitizes TLRs 7 and 8, signaling through adapter proteins, and incites the production of type I interferon [50,51]. Type I interferon is a potent flare that is responsible for the activation of APCs. In the presence of type I interferons, dendritic cells (in the resting state) become active with increased expression of B7-1 (CD80) and B7-2 (CD86) costimulatory molecules, and MHC (major histocompatibility complex) class I/II [50,51]. Type I interferon also has profound effects on cellular immunity; specifically, these interferons directly act on antigen specific CD8 T cells to significantly expand them [52]. In addition to mRNA’s direct effects on innate immunity, it could be leveraged as a tumor specific vaccine for all patients. Since RNA bypasses MHC class restriction, allowing a patient’s cell machinery to process and present their HLA (human leukocyte antigen) specific MHC processed epitopes, it can be used in all patients and not just pre-specified HLA haplotypes (i.e., HLA-A2 restriction for peptide vaccines) [53]. Moreover, RNA is easy to produce, and can be safely stored before reconstitution and patient administration [53].

One drawback of RNA is its fundamental lack of stability, making it difficult to administer ‘naked’ RNA to patients in vivo. Since cancer vaccines must localize to APCs where RNA must be translated, processed, and presented on MHC class I and II molecules, degradation has been a potent barrier for the development of new mRNA technologies. To overcome these limitations, RNA has been administered through direct intranodal injections and has shown promise in early phase studies of refractory cancers [54,55]. RNA might also be stabilized through the addition of untranslated regions [56,57]. While these serve to protect RNA from degradation prior to reaching its intended target, RNA could still be degraded inside the cell [46], and within its compartments [58]. Alternatively, delivery vehicles have been developed to deliver RNA and to protect it from degradation before and after cell transfection. 

## 3. Liposomal Delivery Vehicles

Nanocarriers have been developed as delivery vehicles for RNA, however novel designs remain mired with unknown in vivo reactivity, requiring rigorous preclinical toxicity studies in large animal models [59]. Given the complexity and cost of these studies, translatable lipid-nanoparticles (NPs) have been developed as delivery vehicles to protect and deliver RNA to intended cell types in vivo. Lipid-NPs can be developed with solid cores for the development of controlled-release agents (i.e., solid lipid nanoparticles or SLPs); they can also be developed with aqueous cores as nanoliposomes [60,61]. Given the large amount of preclinical toxicology data for some nanoliposomes, these agents may provide a more straightforward path for development as RNA delivery vehicles in human clinical studies [61]. Nanoliposomes benefit from an abundance of preclinical toxicity data, promising safety data, and application in early phase studies [61,62,63,64]. Although they have been developed primarily in the context of drug and gene delivery, liposomes may fail in these contexts due to inadequate localization to target sites [65]. In the systemic circulation, liposomes tend to localize to reticuloendothelial organs such as the liver and spleen, and while not ideal target sites for a drug or gene therapy, these are more optimal locations for a cancer vaccine to induce APC transfection and T cell priming [65]. These organs are imbued with antigen presenting cells (i.e., splenic macrophages, dendritic cells, Kupffer cells) that can take up RNA and incite an activated T cell response against the desired epitopes encoded by the mRNA [66,67]. Based on these features, RNA-loaded liposomes have been developed as cancer immunotherapeutic agents with promising results in both pre-clinical models and clinical trials [68,69]. These RNA-liposomes are often administered intravenously to incite robust amounts of inflammatory cytokines before the production of antigen specific immunity [68,69]. Given their capacity for surface and core modifications, liposomes have tremendous versatility. They can be embedded with multiple mRNAs encoding for immunomodulatory cytokines and offer a promising, simpler alternative to cell therapy vaccines (i.e., dendritic cell vaccines) [68,69]. Since they are composed of nucleic acids that are condensed in a nanocarrier, these vaccines appear to mimic viremia through the induction of a characteristic type I interferon signature; however, instead of inducing an anti-viral response against viral antigens, RNA-liposomes redirect innate and adaptive host immunity against mRNA antigenic material that is expressed by the tumor [69]. 

**Figure 1:**
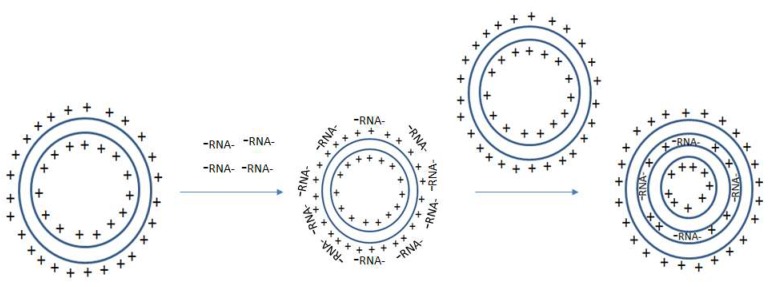
Proposed Schema for RNA-liposome encapsulation: A bilamellar lipid particle with a positively charged surface and inner core electrostatically interact with negatively charged RNA. Negatively charged RNA coats the surface of the bilamellar lipid particle, which electrostatically interacts with a new positively charged bilamellar lipid particle. The new positively charged bilamellar lipid particle complexes with the original RNA coated bilamellar lipid particle. This forms a multilamellar particle where the RNA is trapped between the two layers of the lipid particles.

The type of immune response that is elicited by liposomes is often affected by the lipid material’s surface properties. Liposomes may be polar or non-polar [70]. Polar lipids are effective agents for mRNA transfection and are typically composed of hydrophilic head groups that are attached to linker bonds connected with non-polar tails [70]. The non-polar tails from separate molecules join one another so that the positively charged hydrophilic heads (repelled by one another) face along opposite sides [70]. As more liposomes join together, these NPs form micelle-like structures that are composed of a positively charged outer surface, a positively charged inner core, and a lipid layer in between [70]. Positively charged liposomes can simply be mixed with negatively charged nucleic acids (i.e., RNA) for the formation of RNA-liposomes. A proposed schema for RNA-liposome complexation is shown in Figure 1. Briefly after the addition of positively charged liposomes to negatively charged RNA, electrostatic interactions dominate. Negatively charged RNA adheres to the surface of the particle creating a net negative charge that may be enveloped by another positively charged particle. This effectively traps the nucleic acid RNAs between the lipid envelopes, protecting them from degradation. This process can be repeated so that multiple layers of mRNA coat successive outer layers before being enveloped by new liposomes, forming multi-lamellar vesicles. These multi-lamellar vesicles can maintain their small sizes due to the internal electrostatic interactions that keep the particle tightly packaged. 

The electrostatic interactions between RNA and liposomes could have unintended effects in vivo. Small positively charged particles might aggregate, forming larger particles that could adhere to plasma proteins in the bloodstream [58]. These interactions may affect particle uptake and distribution. For example, larger aggregate particles could be preferentially taken up by macrophages through phagocytosis, while smaller particles might be endocytosed by parenchymal cells or pinocytosed by dendritic cells [58]. For these reasons, in vitro effects of RNA-liposomes may not be truly representative of their in vivo effects and interactions. While standardizing liposome batches may help to control size and in vivo effects (i.e., distribution), heterogeneity is often expected. Consequently, characterization studies (i.e. liposome size, charge, and shape) are necessary. Slight changes in these surface properties may have significant effects on biologic activity, including transfection efficiency, intracellular processing/trafficking, and toxicity profile. 

Although RNA loaded liposomes have been used in several preclinical studies and appear to have a promising safety profile, adverse effects have been reported. Positively charged particles have been shown to mediate transaminitis in preclinical studies and may elicit apoptosis of transfected cells [71,72]. Much of these effects, however, could be secondary to their immune adjuvant effects. Positively charged liposomes elicit the production of pro-inflammatory cytokines and stimulate TLRs [71,72]. While these effects would be unattractive for drug, gene, or siRNA delivery vehicles, these effects may be advantageous for a cancer immunotherapeutic agent. Despite these risks, nanoliposomes bypass systemic toxicities through rapid clearance from circulation [71,72]. This rapid clearance mitigates the risks of lipid cargo (i.e., chemotherapeutic agents) while enhancing distribution of the payload to end organs. As new technologies are developed, the risks of liposome delivery vehicles must be weighed against their benefits. For some liposomes (i.e., DOTAP), there exists a boon of safety data in human clinical trials that can be leveraged for the development of new agents.

## 4. Cationic Lipid DOTAP in Human Trials 

DOTAP is one of the first liposomes to be developed and has been under investigation for decades [73]. As such, there is a plethora of preclinical and clinical toxicity data in human gene and drug delivery trials [73]. DOTAP is composed of an amine head group that is attached to linker bonds that connect the head group to hydrophophic tails [73]. In one of the first clinical studies, DOTAP was utilized to deliver the CFTR gene, which is abnormal/deficient in patients with cystic fibrosis (CF), to the lung parenchyma of CF patients [62]. This was accomplished through the delivery of a vector encoding for the CFTR gene that was encapsulated into DOTAP liposomes [62]. The agent was administered by intranasal injection and resulted in transgene expression [62]. DOTAP liposomes have also been formulated into nanoscale particles for intratumoral drug delivery [63,64]. Since malignancies have leaky vasculature, DOTAP liposomes can be harnessed for preferential intratumoral delivery and enhanced permeability and retention (EPR) therein [63,64]. Although chemotherapeutic agents such as paclitaxel are associated with systemic toxicities (precluding higher chemotherapeutic doses that may be necessary to treat patients with refractory cancers), encapsulation of paclitaxel into DOTAP liposomes may increase intratumoral delivery [63,64]. These liposomes (under the trade name EndoTAG-1) have been studied in phase I/II studies for patients with refractory tumors (i.e., metastatic liver disease, head and neck squamous cell carcinoma, and HER2 negative breast cancer) and appear to be safe and tolerable [63,64,74]. In a phase II study for patients with triple negative breast cancer, EndoTAG-1 in combination with paclitaxel, elicited anti-tumor efficacy [74,75]. Based on these promising data, a phase III study is currently underway evaluating the safety and efficacy of EndoTAG-1 in conjunction with paclitaxel and gemcitabine in patients with visceral metastatic triple-negative breast cancer (clinicatrials.gov: NCT03002103), and in phase III trials with gemcitabine for patients with locally advanced/metastatic pancreatic adenocarcinoma (clinicaltrials.gov: NCT03126435). In addition to drug delivery for better EPR effect, DOTAP liposomes have also been utilized as carriers for plasmids and oligonucleotides in human trials. DOTAP liposomes were used as intratumoral delivery vehicles of synthetic deoxyribozyme oligonucleotides in basal cell carcinoma [76]. In patients with basal cell carcinoma, intratumoral delivery of DOTAP (combined with DOPE, a neutral helper lipid to enhance endosomal disruption) complexes encapsulating Dz13 deoxyribozyme targeting c-jun mRNA were well-tolerated [76]. DOTAP liposomes were also utilized to deliver a tumor suppressor gene TUS2/FUS1 that typically becomes inactive during lung cancer evolution and propagation [77]. In a phase I study, DOTAP liposomes, formulated with cholesterol encapsulating the TUSC2 plasmid, were administered systemically to patients with recurrent and/or metastatic (i.e. small cell or non-small cell) lung cancer [77]. In this study, the DOTAP liposomes were safely administered and displayed anti-tumor activity [77]. These nanoliposomes are currently under investigation in combination with erlotinib in phase I/II studies (clinicaltrials.gov: NCT01455389). In addition to DOTAP, examples of other cationic lipids that have been translated into human clinical trials are indicated in Table 1 [61]. These cationic lipids can be mono- or polycationic [78], and cholesterol can be embedded with the lipid construct [78]. Polycationic liposomes may enhance transfection efficiency, however increasing charge may also increase inflammatory toxicity [79]. Similarly, cholesterol (chol) enhances liposome stability, but it may also increase complement activation at higher membrane lipid amounts (>70%) [80].

## 5. RNA-Loaded Liposomes

Due to the proof of concept studies demonstrating the safety of liposomes like DOTAP in first-in-human trial application, the translation of new RNA-liposome technologies are not mired with as many regulatory hurdles. Several RNA-liposomes have already been investigated in human trials, including RNA interfering agents that have been safely used in phase I studies. Small interfering RNAs (siRNAs) that inhibit vascular endothelial protein kinase N3 encapsulated in cationic liposomes for solid tumor disruption were well tolerated in a human phase I study [81,82]; this agent is currently being investigated in a phase IB/IIa study with gemcitabine in patients with pancreatic adenocarcinoma (clinicaltrials.gov: NCT01808638). In a separate clinical trial, EphA2 is being targeted with siRNA that is encapsulated in neutral DOPC liposomes for patients with advanced solid tumors (clinicaltrials.gov: NCT01591356) [83]. 

While liposomes have predominantly been developed for intratumoral targeting via EPR effect, the development of mRNA-liposomes as cancer immunotherapeutic agents requires localization to APCs for the activation of antigen specific T cell immunity. For mRNA-liposomes, this poses a distinct challenge. After intravenous administration of lipid particles, first pass organs (i.e., the heart and lung) are often the initial sites of transfection [78]. When encountering a cell with a negatively charged cell membrane, positively charged particles may become attracted to the first cells they encounter cells within these organs [78]. As a result, RNA loaded liposomes can transfect parenchymal cells or endothelial cells in these organs as opposed to antigen presenting cells [78]. As such, positively charged liposomes are associated with enhanced transfection efficiency in cells/organs of first pass, making them difficult to direct in vivo. Interestingly, Kranz et al. showed that by decreasing the cumulative charge of mRNA-lipid particles (effectively making them anionic in composition), they could localize mRNA-loaded liposomes preferentially to the spleen [84]. They demonstrated that these liposomes (composed of DOTMA with DOPE) encapsulating mRNA encoding for tumor associated antigens or neo-epitopes localize discretely to the spleen, as opposed to first pass sites such as the heart and lung [84]. Therein, mRNA activates toll like receptor 7 (TLR7) on plasmacytoid dendritic cells for the production of type I interferon and induction of robust anti-tumor activity [84]. Similarly, Broos et al. showed that mRNA encoding for model antigens (i.e., OVAlbumin) and physiologically relevant antigens (i.e., E7 from human papilloma virus, tyrosinase related protein 2) encapsulated in lipofectamine RNAiMAX (at composite anionic zeta potentials) could be systemically administered for transfection of APCs in the spleen and liver, and initiation of an immunologic response [85]. After systemic injection, anionic RNA-liposomes may naturally be repelled from cells in first-pass organs before localization to reticuloendothelial organs which contain leakier vasculature from sinusoidal capillaries. While delivery vehicles are often employed with targeting moieties that may alter a nanomaterial’s safety profile, these data demonstrate that through simple changes to biophysical properties (i.e., charge), particle localization could be directed [84,85]. In this manner, splenic targeting of mRNA-loaded lipid-NPs is an attractive approach for immunotherapeutic immune induction that preserves the safety profile of nanoliposomes [84,85]. RNA-liposomes have also been developed to target APCs in regional lymph nodes after subcutaneous administration [86]. Oberli et al. developed a library of lipid-NPs and identified a target formulation composed of an ionizable lipid, cholesterol, phospholipid, and polyethylene glycol [86]. These particles were shown to localize to regional lymph nodes, elicit cytotoxic CD8 responses and induce of anti-tumor efficacy [86]. 

**Figure 2:**
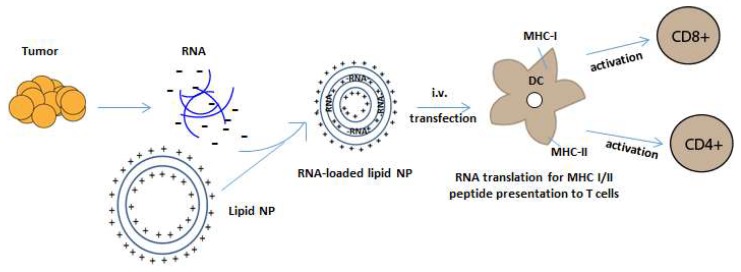
RNA-NP mediated transfection of APCs. Negatively charged tumor mRNA is extracted and amplified from personalized tumor biopsies and is encapsulated into positively charged lipid-NPs. NPs encapsulate RNA through electrostatic interaction and are taken up by dendritic cells in reticuloendothelial organs after systemic administration. The RNA is then translated and processed by an APC’s intracellular machinery for the presentation of peptides onto MHC class I and II molecules, which activate CD4 and CD8+ T cells.

Alternatively, our group has prioritized RNA-liposomal cancer vaccines consisting of personalized tumor derived mRNA (representing a tumor specific transcriptome) that is encapsulated into DOTAP NPs [87]. By extracting total RNA (tRNA) from as little as 500 biopsied cells, these personalized RNA-liposomes can be generated against malignancies (i.e., brain tumors) that are difficult to access surgically. While tRNA contains ribosomal and transfer RNA, we can generate a cDNA library through RT-PCR on mRNA present from the initial tRNA extraction. This cDNA may then be in vitro transcribed and amplified for copious generation of mRNA, representing a tumor-specific transcriptome. By simply mixing positively charged liposomes with negatively charged RNA, NPs become complexed with mRNA for loading of dendritic cells or APCs with tumor antigens (Figure 2). 

Though these RNA-liposomes are not targeted to a specific organ (i.e., the spleen), the systemic release of type I interferon from in vivo transfected cells mediates activation of APCs throughout lymphoid compartments [87]. We showed in a murine adoptive cellular therapy model for high-grade glioma that these RNA-loaded nanoliposomes may be used in place of dendritic cells in the expansion of antigen specific T cells and induction of therapeutic anti-tumor efficacy [87]. 

Unlike synthetic RNA manufacturing (i.e., neoantigens), personalized tumor mRNA loaded liposomes allow APCs to process and present whole tumor transcriptome. Although this might induce autoimmunity, T cell receptors (TCRs) recognizing self-antigens should have been negatively selected by the thymus and are not expected to be present on autologous T cells. Consequently, TCRs recognizing the most foreign/immunogenic tumor epitopes should be activated, allowing the immune system to decide the best tumor targets. This strategy forgoes MHC binding prediction algorithms and other methods (mass spectroscopy) that are necessary to detect the most immunogenic tumor epitopes. Despite these advantages, tracking antigen specific immunity in vivo is challenging when the immunoreactive epitope is unclear. 

To more directly target tumor antigens using T cells, APCs may be bypassed through bispecific T cell engagers (BiTEs) and chimeric antigen receptor (CAR) modified T cells [88,89]. BiTEs are composed of single chain variable fragment regions from two antibodies that are connected by a linker; one scvFr is directed against CD3 and the other is directed against a surface tumor antigen of interest [88]. This technology brings CD3 T cells into contact with tumor cells for induction of T cell directed killing of tumor targets [88]. Since BiTEs may be encumbered by developmental complexities, RNA encoding BiTEs in lipid particles have been used as promising alternatives [90]. RNA liposomes encoding for BiTEs were shown to be translated into functional proteins and may hasten growth and application of BiTE technologies [90]. Similarly, liposomes may enhance CAR T cell technologies. CAR modified T cells imbue the cytotoxicity of a T cell with the specificity of an antibody, bypassing their need for antigen presentation and TCR mediated activation [89]. While this platform has shown promise against hematological malignancies, it remains limited in solid tumors due to the intratumoral microenvironment’s suppressive effects on CAR T cell activity [91]. Tumor targeted liposomes, however, loaded in part with PI3 kinase inhibitors were shown to remodel the solid tumor microenvironment, enabling CAR T cell technologies to mediate potent anti-tumor efficacy against preclinical solid tumor malignancies [91]. These liposomes may assist the delivery of CARs to tumor tissue for intratumoral immune reprogramming.

While still in the early stages, RNA-liposomes have been tested in human clinical studies. A dose-escalation study explored the safety and activity of tetravalent RNA-lipoplex vaccines (targeting four tumor-associated antigens) in patients with advanced melanomas (clinicaltrials.gov: NCT02410733). Aside from transitory flu-like illnesses, the vaccines have been safe and well tolerated with activity based on the induction of IP-10 and type I interferon [84,92]. More personalized RNA-liposomal cancer vaccines are currently being investigated in multi-institutional human clinical trials [92]. Phase 1a/1b studies are underway, investigating personalized RNA-loaded liposomes in combination with immune checkpoint inhibitors, atezolizumab (mAbs targeting PD-L1), for patients with refractory solid tumors (clinicaltrials.gov: NCT03289962).

## 6. Conclusions

While innovative NP designs continue to be constrained by toxicology concerns and unknown in vivo effects, well characterized lipid-NPs have been prioritized as RNA delivery vehicles. Liposomes are composed of biocompatible materials and appear to have favorable safety profiles. Instead of engineering these lipid backbones with targeting moieties, changes to the charge ratio (of negatively charged RNA to positively charged liposome) and lipid composition can hone these particles to lymphoid organs. Other translational modifications that preserve these well-characterized lipid backbones (i.e., DOPE, cholesterol, poly-ethylene-glycol (PEG)) may increase biocompatibility and liposome circulation time for an intratumoral EPR effect. RNA-liposomes are now in human clinical trials and have shown promising early results. While cancer vaccines have traditionally suffered from inadequate immunogenicity in therapeutic settings, RNA-nanoliposomes are expected to harness multiple arms of the immune system for expeditious and effective anti-tumor immunity. 

## Figures and Tables

**Table 1:** Examples of cationic lipids used in clinical trials [61].

Cationic Lipid	Charge	Cholesterol	Advantages	Disadvantages
DOTAP	Monocationic	Non-embedded	Well-studied; promising safety profile	Decreased targeted localization
DOTMA	Monocationic	Non-embedded		
DMRIE-Chol	Monocationic	Embedded	Increased liposome stability [80]	Complement activation with increasing lipid membrane cholesterol (>70%) [80]
DOTIM-Chol	Monocationic	Embedded		
EDMPC-Chol	Monocationic	Embedded		
DC-Chol	Monocationic	Embedded		
DOSPER	Polycationic	Non-embedded	Enhanced transfection efficiency	Increased toxicity/inflammation
DOSPA	Polycationic	Non-embedded		
GL-67	Polycationic	Cholesteryl-Embedded		
